# Monitoring of internal load, sleep, and well-being in relation to injury and illness in professional basketball

**DOI:** 10.1186/s13102-026-01663-3

**Published:** 2026-03-26

**Authors:** Jakob Burger, Richard Latzel, Thomas Voit, Lynn Matits, Othmar Moser, Alexander-Stephan Henze

**Affiliations:** 1 Division of Exercise Physiology and Metabolism, University of Bayreuth, Bayreuth, Germany; 2 Faculty of Applied Natural Sciences and Industrial Engineering, Deggendorf Institute of Technology, Deggendorf, Germany; 3 Division of Clinical and Biological Psychology, Institute of Education and Psychology, Ulm University, Ulm, Germany; 4 Sports and Rehabilitation Medicine, University Hospital Ulm, Leimgrubenweg 14, Ulm, 89075 Germany; 5 Interdisciplinary Metabolic Medicine Research Group, Division of Endocrinology and Diabetology, Medical University of Graz, Graz, Austria

**Keywords:** Team sports, Load management, Psychological well-being, Athletic injuries, Primary prevention

## Abstract

**Background:**

Athlete health is essential for sustained performance in professional sport, with injuries and illnesses limiting both individual and team success. Although athlete monitoring is widely used to support load and recovery management and reduce injury risk, evidence linking training load, well-being and health outcomes in professional basketball is inconsistent. Holistic monitoring approaches may enhance the understanding of load-related health dynamics. Therefore, this study aimed to investigate the associations between internal load, sleep, and physical and mental well-being and the occurrence of injury and illness in professional male basketball players across a competitive season.

**Methods:**

In this prospective observational study (German Clinical Trials Register ID: DRKS00032713; registered 19 October 2023), 16 professional male basketball players were monitored over a 31-week competitive season using a software application (Prevention Management Tool, PMT) provided by the German Accident Insurance for professional team sports. Structured injury and illness surveillance was conducted throughout the study period. Internal load was assessed 30 min after each training session and match using the session rating of perceived exertion (sRPE) method. In addition to sleep duration, PMT-integrated athlete self-reported measures collected each morning assessed sleep quality, physical and mental well-being using 11-point Likert scales (0–10). Linear mixed-effects models were used to investigate the associations between internal load, sleep and well-being and the occurrence of injury and illness.

**Results:**

A total of 3,454 athlete records were included in the analyses, corresponding to a completion rate of 88.9%. Mean weekly internal load derived from sRPE was 3,873 ± 1,524 arbitrary units. Mean daily sleep duration was 8.16 ± 1.17 h. Mean scores for sleep quality, physical well-being, and mental well-being were 7.59 ± 1.34, 7.78 ± 1.27, and 7.76 ± 1.28, respectively. The cumulative season incidence rates were 1.31 for acute injuries, 0.50 for overuse injuries, and 2.06 for illnesses. Higher internal load and lower sleep quality were associated with reduced physical and mental well-being. Lower physical well-being was significantly associated with illness occurrence (*P* < .001, *η*²ₚ = 0.03), whereas physical and mental well-being were not associated with injury occurrence.

**Conclusions:**

Higher internal load and lower sleep quality were associated with reduced well-being in professional male basketball players. Decreased physical well-being was associated with illness occurrence, supporting its relevance as a potential early indicator within athlete health monitoring frameworks.

**Supplementary Information:**

The online version contains supplementary material available at 10.1186/s13102-026-01663-3.

## Background

Athlete health is a central pillar for sustained performance and success in professional team sports [1, 2]. Injuries and illnesses remain major barriers to both individual and team performance, leading to reduced player availability and impaired competitive outcomes [2, 3]. Consequently, strengthening athlete health and reducing potentially preventable injuries, illnesses, and other athlete health problems has become a key objective of high-performance sport systems [4–6]. Effective prevention strategies have been shown to reduce injury risk across a range of sports [7, 8]. Accordingly, systematic injury and illness surveillance represents a fundamental component of athlete health protection and evidence-informed prevention strategies [6].

Within this context, athletes are exposed to varying physiological and psychological demands during training and competition, commonly conceptualized as external and internal load [9]. External load reflects the objective physical work performed by an athlete, whereas internal load represents the individual psychophysiological responses elicited by this work, integrating contextual factors such as fatigue and recovery status [9]. In professional basketball, characterized by frequent high-intensity actions and congested competition schedules, combined monitoring of external and internal load is considered best practice [10]. However, particularly in applied settings with limited resources, internal load monitoring remains a practical and widely implemented approach across elite and sub-elite levels [11]. In this regard, the subjective single-item measures *rating of perceived exertion* (RPE) and the *session rating of perceived exertion* (sRPE*)* are the most commonly used methods [12]. The sRPE method has demonstrated validity and reliability across various sports, including basketball [13, 14], and shows meaningful associations with external load parameters [15, 16]. Nevertheless, although relationships between load parameters and injury risk have been investigated in basketball, findings remain inconsistent [17–20], highlighting the complexity of load–health interactions.

Contemporary athlete health research increasingly supports holistic, system-oriented frameworks in which injury and illness are viewed as downstream outcomes of maladaptation resulting from imbalances between load and recovery [21, 22]. Within these frameworks, athlete well-being is considered a key recovery-stress-related construct that may mediate or modify the effects of training and competition load on health outcomes [5]. Adequate sleep duration and quality are essential for physiological and mental recovery [23, 24], and insufficient sleep has been associated with increased injury susceptibility in basketball players [25]. Although the relationship between athlete well-being and in-season injury risk has been examined previously, results remain inconclusive [24–26].

Advances in athlete monitoring systems (AMS) and decision-support strategies have facilitated the integration of multiple data streams, allowing practitioners to contextualize load data alongside recovery and well-being indicators [27]. In applied settings, athlete self-reported measures (ASRMs) remain widely used due to their feasibility, low cost, and demonstrated validity [9, 13, 28]. However, reliance on subjective measures in isolation presents challenges, including individual response bias and contextual variability [27, 28]. Recent research therefore emphasizes the importance of embedding subjective monitoring tools within broader monitoring frameworks rather than using single metrics as standalone predictor of injury or illness [27]. In this context, the Verwaltungs-Berufsgenossenschaft (VBG), the statutory accident insurance provider for professional team sports in Germany, has integrated several ASRMs assessing internal load, sleep quality, and physical and mental well-being into its AMS, the “Prevention Management Tool” (PMT).

Despite growing interest in such holistic AMS approaches, evidence linking internal load, sleep, well-being, and health-related outcomes in professional basketball remains limited and inconsistent [25, 26]. While previous studies have examined selected components of this framework, few have simultaneously investigated internal load, sleep, and distinct dimensions of athlete well-being in relation to both injury and illness across a full competitive season. Moreover, illness outcomes remain underrepresented in basketball research, despite the relevance for player availability and performance.

Therefore, the aim of this prospective observational study was to (i) examine the effects of internal load, sleep duration, and sleep quality on physical and mental well-being as assessed ASRMs integrated into the official AMS, and (ii) explore associations between athlete well-being and the occurrence of injury and illness in professional male basketball players across a competitive season. By integrating routinely collected monitoring data within an applied AMS, this study seeks to provide practice-relevant evidence to inform load management, recovery strategies, and health-related decision-making in professional basketball settings.

## Methods

### Study design

This prospective longitudinal observational study collected daily athlete monitoring data alongside injury and illness surveillance data over the 2023/24 competitive season (August 2023 to March 2024; 31 weeks of data collection) in a professional German basketball team. All participants, or their legal representatives in the case of adolescents, provided written informed consent prior to participation. The study was conducted in accordance with the Declaration of Helsinki, registered in the German Clinical Trials Register (ID: DRKS00032713, Date: 19/10/2023), and approved by the local ethics committee (University of Bayreuth, approval no. 23–027).

Reporting of this study followed the *International Olympic Committee* (IOC) recommendations, including the *Strengthening the Reporting of Observational Studies in Epidemiology* (STROBE) *Extension for Sport Injury and Illness Surveillance* (STROBE-IIS) guidelines [6].

### Participants

A total of 19 professional male basketball players of a single team competing in the third tier of the German professional league system were initially recruited for participation in the study. Eligible participants (i) competed at the professional level (Tier 3) [29], (ii) were aged ≥ 16 years, (iii) having successfully completed a pre-competition medical assessment (PCMA) at baseline, and (iv) participated in at least 80% of the training sessions and matches during the study period. Players were excluded if they were concurrently enrolled in another study or had any medical condition that could interfere with study participation or interpretation of results. Participants were also excluded from the final analysis in cases of insufficient compliance with the study procedures or if they sustained a time-loss injury outside of regular basketball practice or competition. Of the 19 recruited players, 16 met al.l inclusion criteria and were included in the final analysis.

### Training schedule

**Fig. 1:**
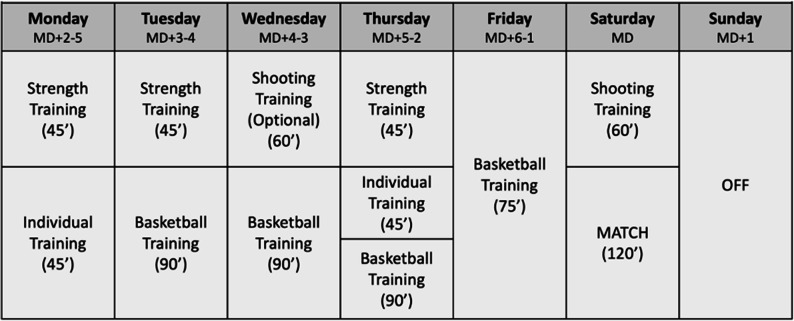
In-season weekly micro cycle. Idealized in-season week (MD = X format), showing a typical structure of strength, individual, and basketball-specific training sessions, with match play scheduled on Saturday (MD) and a rest day on Sunday (MD + 1). Abbreviations: MD match day, ' minutes

As the present study followed an exclusively observational design, neither the competition calendar nor the training schedule was modified by the research team. The weekly in-season schedule typically consisted of six training days and one designated rest day (Sunday). Training content varied across the week and included structured strength and conditioning session, individual skill development, and team-based practice. Training volume was periodized by the coaching staff according to a standard competitive microcycle. The competitive fixture was scheduled on Saturday and comprised a 120-minute official match, preceded by a preparatory warm-up and shooting session. A full rest day was implemented on Sunday to facilitate post-match recovery. A representative in-season microcycle is presented in Fig. 1.

### Injury and illness surveillance

In accordance with the IOC consensus statement on methods for recording and reporting epidemiological data in sport [6], a health problem was defined as any condition resulting in a deviation from full health and in an inability to participate in the team’s regular, scheduled training routine. To systematically document health problems, participants were instructed to self-report any new pain or other symptoms using the *Prevention Management Tool* (PMT) [30], a cost-free web-based application provided by the *Verwaltungs-Berufsgenossenschaft* (VBG), the official accident insurance provider for athletes in German professional team sports. Following a report, the strength and conditioning coach conducted a structured follow-up assessment to further evaluate the health problem and referred the player to the team physician for medical evaluation. The team physician classified each health problem as either injury or illness, with injuries further categorized as acute or overuse. Only injuries and illnesses leading to the inability to participate in at least one training session or match included in the final analysis.

Prevalence was defined as the proportion of players who sustained at least one time-loss injury or illness during the study period relative to the total number of participants. Incidence was calculated as the average number of time-loss injury or illness incidents per player over the entire study period (cumulative season incidence).

### Monitoring of internal load, sleep, and well-being

Daily monitoring of internal load, sleep, and physical and mental well-being was conducted using self-report measures provided by the VBG in their PMT.

The sRPE method was used to assess internal load [31]. Therefore, players reported the individual duration of each training session or playing time in matches and their RPE from the end of the session up to 120 min thereafter. sRPE was calculated by multiplying session duration by RPE, and daily and weekly internal load were derived by summing sRPE values across all training sessions and matches.

Each morning, players reported their previous night’s sleep duration in hours and rated sleep quality using the non-validated PMT item “How refreshing was your sleep?” on an 11-point Likert scale (0–10). Physical well-being was assessed using the non-validated PMT item “How fit do you feel?”, and mental well-being was assessed using the non-validated PMT item “How do you feel mentally?”, both rated on 11-point Likert scales (0–10).

Prior to data collection, a standardized workshop was conducted with all participants to explain the sRPE method and other PMT items and ensure consistent understanding of the response scales, thereby supporting standardization and compliance.

### Statistical analysis

GraphPad Prism (version 10.3.1, GraphPad Software, LLC) and IBM SPSS Statistics (version 29.0.2.0) was used for statistical analysis. Descriptive data are presented as raw data with mean and standard deviation (SD) for metric variables. Pearson’s correlation coefficient (*ρ*) with 95% confidence interval estimation based on Fisher’s r-to-z transformation with bias adjustment was used for multicollinearity checks.

Due to the hierarchical data structure (repeated measurements clustered within participants), a multilevel analysis was performed to examine the effects of internal load and sleep measures on well-being items. The intraclass correlation coefficient (ICC), calculated as the ratio of the between-subject variance to the sum of the between-subject and within-subject variance derived from the respective random intercept-only models, was ICC = 0.353 for physical well-being and ICC = 0.498 for mental well-being. Linear mixed-effects models (LMEMs) with random intercept (participant ID) and random slope and with previous day internal load (as the sum of the sRPE values), sleep quality and sleep duration as fixed effects were calculated separately for both well-being items. Additionally, an interaction effect (daily internal load*sleep quality) was tested to analyze a possible moderation. Separate generalized LMEMs were calculated by using binary logistic regression with physical or mental well-being as a fixed effect and random intercept to examine the effect of these well-being items on the occurrence of injury or illness. The repeated measures covariance structure of the LMEMs was assumed to be first order autoregressive. Full maximum likelihood (ML) method with Satterthwaite approximation was used for parameter estimation. Effect sizes were calculated as partial eta-squared (*η*^2^_p_) with 95% confidence interval (CI) and categorized according to Cohen as small 0.01 ≤ x < 0.06, medium 0.06 ≤ x < 0.14, and large > 0.14 [40]. An α level of 0.05 (2-tailed) was considered significant.

## Results

### Descriptive data

A total of 16 participants were included in the final analysis (age: 18.38 ± 2.04 years; body height 194.3 ± 10.48 cm; body weight 90.62 ± 14.06 kg). Across the study period, 3,454 days were documented, comprising 2,394 days with training or competition, 808 rest days, and 297 health-related absence days. Data completion rate was 88.9%. Missing data were primarily attributable to untimely data entry. Individual completion rates ranged from 81.6% to 96.2%. Participants were exposed to a total of 5,231.5 h of training or match-play). Mean daily training duration was 98.71 ± 65.11 min, irrespective of day type. On training days only, players completed 126.47 ± 43.82 min across 1.65 ± 0.65 sessions.

**Fig. 2:**
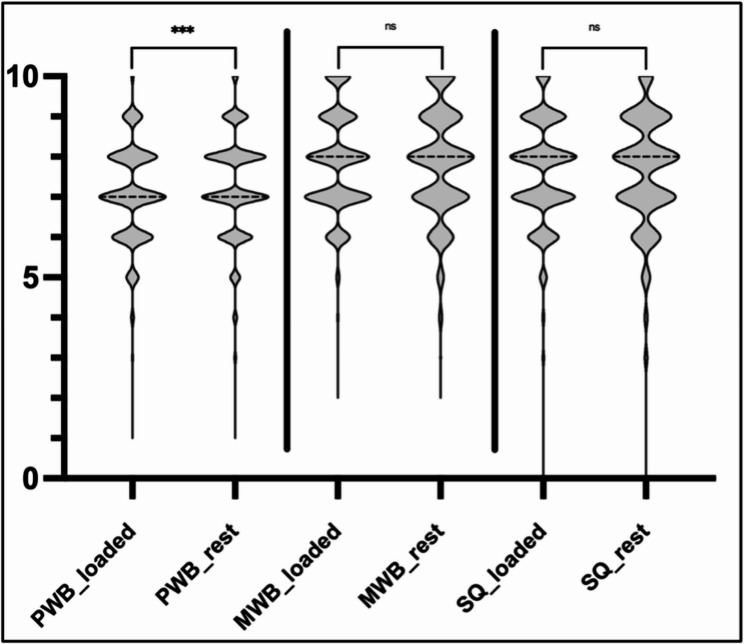
Differences of average well-being and sleep quality between loaded days and rest days. The Y-axis shows the averages (median) of physical well-being (physical well-being), mental well-being (mental well-being), and sleep quality split between loaded days and rest days. Rated on a category ratio-10-scale across the entire 31-week regular season (August 2023 to March 2024). Medians are indicated by black dashed lines, interquartile ranges by white dotted lines. *N* = 16

Mean weekly internal load was 3,873.3 ± 1,524.2 arbitrary units. Average sleep duration was 8.16 ± 1.17 h, with a sleep quality of 7.59 ± 1.34. Physical well-being and mental well-being scores averaged 7.78 ± 1.27 and 7.76 ± 1.28. Differences in physical and mental well-being, and sleep quality between loaded days and rest days across the study period are presented in Fig. 2.

### Correlations between internal load, sleep and well-being measures

Previous-day internal load showed negative associations with subsequent physical and mental well-being, as well as with sleep quality. In contrast, sleep duration was positively correlated with sleep quality, and well-being dimensions were strongly interrelated. Sleep quality also correlated positively with both physical and mental well-being, while sleep duration showed smaller positive associations with well-being and with internal load (see Supplementary Table 1).

### Relationship between internal load, sleep, and physical well-being

On physical well-being, the calculated LMEM showed a highly significant effect of previous-day internal load (b = -0.001 [0.00004], t(2365.26) = -15.1, *P* < .001, *η*^2^_p_ = 0.09 [0.07, 0.11]) and previous-night sleep quality (b = 0.23 [0.01], t(2655.24) = 16.41, *P* < .001, *η*^2^_p_ = 0.09 [0.07, 0.11]). The more complex LMEM with an interaction effect (daily internal load × sleep quality) showed no change in the effect of internal load on physical well-being with sleep quality as a moderator (*P* = .941). Inclusion of the previous night sleep duration as an additional fixed effect did not reach significance for this sleep parameter (*P* = .920).

### Relationship between internal load, sleep, and mental well-being

On mental well-being, the LMEM also showed a highly significant effect of the previous day’s internal load (b = 0.00 [0.00], t(2281.53) = -5.42, *P* < .001, *η*^2^_p_ = 0.01 [0.01, 0.02]) and the previous night’s sleep quality (b = 0.19 [0.01], t(2564.87) = 14.45, *P* < .001, *η*^2^_p_ = 0.08 [0.06, 0.10]). The more complex LMEM with an interaction effect (daily internal load*sleep quality) showed again no change in the effect of internal load on mental well-being with sleep quality as a moderator (*P* = .264). Inclusion of the previous night’s sleep duration as an additional fixed effect did also not reach significance for this sleep parameter (*P* = .066).

### Injury and illness surveillance and associations with well-being measures

The prevalence of health-related absences during the study period was 93.8%, with 15 out of 16 participants experienced at least one absence due to injury, illness, or other athletic health problems. Specifically, 21 acute injuries (50.0% prevalence, cumulative incidence 1.31), 8 overuse injuries (37.5%, 0.50), and 33 cases of illness (93.8%, 2.06) were recorded.

**Fig. 3:**
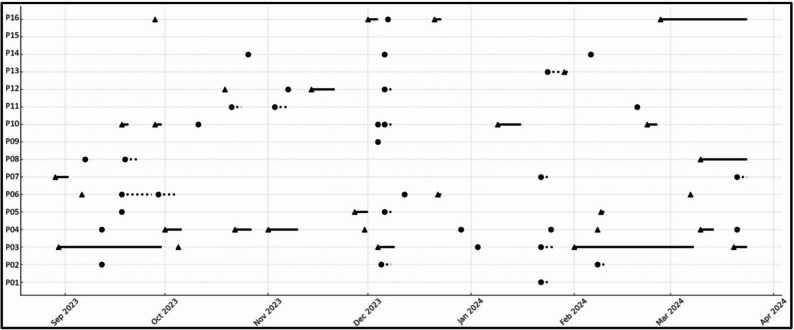
Injury and illness timeline. Circles indicate illness onsets, and dotted lines represent illness periods. Triangles mark injury events, and solid lines represent injury periods. Abbreviation: P participant

Three injuries were classified as season-ending, leading to early withdrawal from the study for the affected athletes. In terms of time loss, 138 days (including rest days) were missed due to acute injuries (mean: 8.63 ± 13.49 days), 67 days due to overuse injuries (mean: 4.38 ± 9.56 days), and 92 days due to illness (mean: 5.75 ± 4.11 days). One participant remained free of illness and injury throughout the entire study period. The participant with the highest number of absence events reported 10 separate occurrences, resulting in a total of 35 days lost. The highest individual time loss was 88 days out of the 216-day observation period. Figure 3 shows individual periods of time loss due to illness and injury.

The calculated generalized LMEM revealed a statistically significant association between lower physical well-being and the occurrence of illness (F = 104.78 (1; 3014), *P* < .001, *η*^2^_p_ = 0.03 [0.03, 0.04]). There were no statistically significant effects of the well-being items on the occurrence of injury (physical well-being: F = 0.25, *P* = .616; mental well-being: F = 0.06, *P* = .800) and mental well-being was not significantly associated with the occurrence of illness (F = 2.26, *P* = .133).

## Discussion

The present study examined the longitudinal relationships between internal load, sleep quality, and physical and mental well-being across a 31-week competitive season in professional basketball players. The main findings were: (i) higher previous day internal load and lower sleep quality were associated with lower subsequent physical and mental well-being; (ii) sleep quality did not moderate load–well-being relationship; and (iii) lower physical well-being was associated with a higher occurrence of illness, but not injury occurrence.

### Internal load and next-day well-being

Previous-day internal load demonstrated small-to-moderate negative correlations with both physical and mental well-being. Linear mixed-effects modelling confirmed these findings, with internal load explaining a meaningful proportion of variance in physical well-being (*η*²ₚ = 0.09) but only a small proportion in mental well-being (*η*²ₚ = 0.01). This association between previous-day training or match load and next-day physical and mental well-being is consistently supported by the literature, including findings from professional basketball cohorts [14, 32–36]. Importantly, the effect was more pronounced for physical than mental well-being. This aligns with the mechanistic expectation that acute external load – reflected in elevated internal load – primarily manifests as musculoskeletal fatigue, soreness, or reduced physical capability and therefore performance [33, 34, 36]. Mental well-being may be more strongly influenced by psychosocial and contextual factors than by training or match load alone [37, 38]. Psychosocial overload occurs when training demands overwhelm coping resources and interact with sport-related and non-sport-related stressors, including inadequate rest, musculoskeletal pain, concerns over capability, and personality variables such as anxiety, mood problems, and maladaptive coping [5, 37, 38]. The multifactorial nature of mental well-being is further evidenced by the finding that competitive stress, organizational demands (i.e., coaching behaviour, team culture, logistic, ), and personal factors all contribute additively to mental health outcomes in athletes [39].

### Role of sleep: quality versus duration

Sleep quality demonstrated a significant effect on both physical and mental well-being (*η*^2^_p_ = 0.08–0.09), whereas sleep duration did not contribute additional explanatory value in the LMEMs when both variables were included simultaneously. Although sleep duration showed positive correlations with well-being items, its effect was no longer significant after adjustment for sleep quality. This distinction is clinically relevant and aligns with findings from basketball and other team sport populations [40, 41]. Sleep quality likely captures perceived restoration, sleep continuity, and subjective recovery status, which may be more sensitive to training-induced fatigue than total time spend in bed [40–42]. From an applied perspective, monitoring sleep quality may therefore provide more actionable information for load management and recovery optimization than duration alone [42].

Furthermore, sleep quality did not moderate the negative effect of previous-day internal load on next-day well-being. The impact of internal load on well-being remained significant irrespective of sleep quality, suggesting independent and additive effects of internal load and sleep quality on athlete well-being [43]. These findings emphasize the importance of systematically monitoring and optimizing sleep quality, rather than focusing solely on sleep duration, to support both physical and mental well-being in professional basketball players [40–44]. Accordingly, the use of a single-item sleep quality measure, as applied in the present study, appears both appropriate and practically feasible in high-performance settings [43].

### Well-being, injury, and illness

The injury and illness surveillance data revealed a high prevalence of illness (93.8%) and substantial injury burden, including season-ending cases. The injury prevalence in the present study (62.5%) was comparable to the data reported in the VBG Sports Report for professional team sports (66.7%) [44].

Lower physical well-being was significantly associated with illness occurrence but not with injury. The association between reduced physical well-being and illness may reflect early prodromal symptoms – such as fatigue, malaise, or reduced vigor – preceding clinically manifest illness. This finding supports the utility of daily subjective monitoring as a low-cost screening tool for impeding health disturbances [45].

In contrast, neither physical nor mental well-being was associated with injury occurrence. This may indicate that, in this cohort, injury risk was more strongly linked to cumulative load due to competitive congestion, contact exposure, or other extrinsic factors (i.e., playing role) rather than short-term fluctuations in well-being [46]. Alternatively, the relatively low number of injury events may have limited statistical power to detect associations [47]. In addition, a potential ceiling effect may be apparent due to the limited sensitivity of the non-validated well-being items that could have reduced the variability necessary to detect stronger associations with injury occurrence.

### Practical implications

From an applied perspective, implementing low-cost athlete self-reported measures into a comprehensive AMS provides meaningful insight into internal load, sleep quality, and athlete well-being. In particular, physical well-being scores may assist in identifying increased susceptibility to illness. However, acute fluctuations in well-being alone may be insufficient to predict injury risk in professional basketball and comparable team sport settings.

### Strengths and limitations

A major strength of the present study is the daily monitoring across an entire competitive season with a high data completion rate. The use of LMEMs appropriately accounted for repeated measures within athletes.

However, this study has several limitations. All sleep and well-being measures integrated into the official AMS of the German Accident Insurance (VBG) for professional team sport athletes were self-reported and therefore subject to reporting bias. In addition, the non-validated ASRMs may have limited sensitivity for subtle changes in sleep quality or well-being, which affects comparability with previous studies in professional basketball. Furthermore, external load metrics and some important contextual factors were not monitored additionally. Another major limitation is the small sample size related to the single team design, which restricts the validity and generalizability of these findings.

## Conclusions

Across a full competitive season in professional basketball players, higher previous-day internal load was associated with reduced next-day physical and mental well-being, while sleep quality – but not sleep duration – was positively related to well-being. Moreover, lower physical well-being was linked to illness occurrence but not injury. Despite inherent limitations related to the single team-design and reliance on self-reported measures, these findings highlight the practical value of integrating internal load monitoring with subjective well-being to inform evidence-based load management strategies and thereby enhance athlete health in professional basketball and other team sport settings.

## Supplementary Information

Supplementary Material 1. Table S1: Correlation matrix of physical well-being, sleep measures, and previous-day internal load.

## Data Availability

The datasets generated and/or analyzed in the current study are not publicly available but are available from the corresponding author on reasonable request.

## Abbreviations

AMS: Athlete Monitoring System
AU: Arbitrary Units
CI: Confidence Interval
CR: Category Ratio
ICC: Intraclass Correlation Coefficient
LMEM: Linear Mixed Effects Model
ML: Maximum Likelihood
NRS: Numerical Rating Scale
PCMA: Pre-Competition Medical Assessment
PMT: Prevention Management Tool
RPE: Rating of Perceived Exertion
S&C: Strength and Conditioning
SD: Standard Deviation
VBG: Verwaltungs-Berufsgenossenschaft
