# Supplementary material for: Monitoring of internal load, sleep, and well-being in relation to injury and illness in professional basketball
Source: BMC Sports Sci Med Rehabil. 2026 Mar 26;18:177. doi: 10.1186/s13102-026-01663-3 (PMC13064061; doi:10.1186/s13102-026-01663-3)
Supplement: Supplementary file 1 — Supplementary Material 1. Table S1: Correlation matrix of physical well-being, sleep measures, and previous-day internal load. [file 13102_2026_1663_MOESM1_ESM.docx]

**Supplementary Tab. 1:** **Correlation matrix of physical well-being, sleep measures, and previous-day internal load.**

|  | **Physical Well-Being** | **Mental Well-Being** | **Sleep Duration** | **Sleep  Quality** | **Daily Load (AU) t-1** |
| --- | --- | --- | --- | --- | --- |
| **Physical Well-Being** |  | 0.508; <0.001^***^ | 0.086; <0.001^***^ | 0.482; <0.001^***^ | -0.235; <0.001^***^ |
| **Mental Well-Being** | 0.508; <0.001^***^ |  | 0.063; <0.001^***^ | 0.384; <0.001*** | -0.056;  0.002^**^ |
| **Sleep Duration** | 0.086; <0.001^***^ | 0.063; <0.001^***^ |  | 0.349; <0.001*** | 0.047;  0.013^*^ |
| **Sleep  Quality** | 0.482; <0.001^***^ | 0.384; <0.001^***^ |  |  | -0.075; <0.001^***^ |
| **Daily Load (AU) t-1** | -0.235; <0.001^***^ | -0.056; 0.002^**^ | 0.047; 0.013^*^ | -0.075; <0.001^***^ |  |

Legend: Pearson correlation coefficients (r) with corresponding p-values between physical well-being, mental well-being, sleep duration, sleep quality, and previous-day internal load (Daily Load t-1). Significance levels are indicated as p < .05 (*), p < .01 (**), p > .001 (***).
